# Effects of thyroid cystectomy for primary hyperparathyroidism on immune function

**DOI:** 10.12669/pjms.321.8928

**Published:** 2016

**Authors:** Xiangdang Yin, Liang Hu, Xiaochun Wang

**Affiliations:** 1 Xiangdang Yin, Department of Oral-Maxillofacial-Thyroid Oncosurgery, Jilin Cancer Hospital, Changchun 130001, China; 2 Liang Hu, First Department of Respiratory Diseases, Children’s Hospital of Changchun, Changchun 130051, China; 3 Xiaochun Wang, Department of Oral-Maxillofacial-Thyroid Oncosurgery, Jilin Cancer Hospital, Changchun 130001, China

**Keywords:** Endoscopic thyroidectomy, Primary hyperparathyroidism, Immune function, Thyroid hormone

## Abstract

**Objective:**

To evaluate the effects of thyroid cystectomy for primary hyperparathyroidism on immune function.

**Methods:**

Ninety-two patients with parathyroid cysts complicated with primary hyperparathyroidism were randomly divided into a treatment group and a control group (n=46). The treatment group received endoscopic thyroidectomy through the anterior chest wall via the areolar approach, and the control group was treated with conventional open thyroidectomy.

**Results:**

The two groups had similar immune function indices as well as thyroid hormone, serum calcium and phosphorus levels before surgery (P>0.05). After surgery, FT3 and FT4 levels significantly increased in both groups, whereas that of TSH significantly decreased (P<0.05). The levels of the two groups differed significantly on the postoperative 5th day (P<0.05). NK%, CD3+%, CD4+% and CD8+%, which significantly fluctuated on the postoperative 1st day in both groups (P<0.05), were basically recovered on the postoperative 5th day in the treatment group that had significantly different outcomes from those of the control group (P<0.05). On the postoperative 1st and 5th days, the treatment group had significantly lower serum calcium level and significantly higher serum phosphorus level than those of the control group (P<0.05). The surgeries were successfully performed for all patients. During three months of follow-up, the treatment group was significantly less prone to complications such as surgical site infection, recurrent laryngeal nerve injury, parathyroid crisis and hoarseness than the control group (P<0.05).

**Conclusion:**

For treatment of primary hyperparathyroidism, endoscopic thyroidectomy through the anterior chest wall via the areolar approach decreased the incidence rate of complications, as well as promoted the recovery of serum calcium and phosphorous levels, probably by only mildly affecting immune function and thyroid hormone levels.

## INTRODUCTION

Primary hyperparathyroidism, as a common, frequently occurring disease in clinical practice, mainly endangers young females. Particularly, this disease may be induced by parathyroid cysts, clinically manifested as high serum calcium level, bone lesions, urinary tract stones, etc.^1^ Specific clinical symptoms of primary hyperparathyroidism include pathological changes of the digestive, nervous, skeletal and urinary systems and skin, which may be misdiagnosed as rheumatic and rheumatoid arthritis.^2^,^3^ About 5% of adult parathyroid cysts can be found through palpation, with most of them, although benign, surgically treated due to subjective symptoms of discomfort or anxiety of malignant transformation.^4^

Approximately 2% of thyroid malignancies are developed from benign thyroid diseases which, as suggested by previous evidence, have the tendency to carcinomatous changes.^5^,^6^ Conventional open thyroidectomy has been applied for over one hundred years and improved gradually.^7^ On the other hand, totally endoscopic thyroid surgery is now available for thyroid cystectomy.^8^ Endoscopic thyroidectomy via the areolar approach is conducive to early postoperative recovery by minimizing surgical sites and hiding them distant from the neck as well as by mildly burdening organs and systems.^9^ Meanwhile, immune function is bound to be disordered after surgery as a special form of trauma. Many immune cells and liquid media are involved in early inflammatory response, forming stress response and leading to poor prognosis as a result.^10^,^11^ In this study, we evaluated the effects of two methods for thyroid cystectomy on the immune function of patients with primary hyperparathyroidism.

## METHODS

### Subjects

Ninety-two patients with parathyroid cysts complicated with primary hyperparathyroidism who were treated in our hospital from August 2007 to July 2013 were selected. This study has been approved by the ethics committee of our hospital.

### Inclusion criteria

Pathologically diagnoses as parathyroid adenomas, accompanied by symptoms of hyperparathyroidism; unilateral disease; without hoarseness, irritating cough while drinking water, or dysphagia; without history of breast surgeries or radiation; with surgical indications (nodules had clear boundary and smooth surface, which freely moved along with swallowing); without surgical contraindications; with consent form.

### Exclusion criteria

Thyroid cysts were diametered over 5cm; with heart, lung, liver, kidney dysfunctions, autoimmune diseases, or other diseases that affected immune function; patients who received blood transfusion, surgery or hormone therapy within three months; diagnosed as thyroid cancer or thyroiditis after surgery. The patients were randomly divided into a treatment group and a control group (n=46) and their gender ratio, age, cyst position, cyst nature, disease course and body mass index (BMI) were similar (P>0.05) (Table-I).

**Table-I:** Baseline data.

Index	Treatment group (n=46)	Control group (n=46)	t or χ^2^	P
Gender (female/male)	26/20	25/21	0.045	>0.05
Age (year)	48.33±5.92	48.24±6.09	0.183	>0.05
Cyst position (left/right)	28/18	27/19	0.078	>0.05
Cyst nature (cystic mass/solid mass)	30/16	31/15	0.098	>0.05
Disease course (year)	2.76±0.45	2.77±0.56	0.013	>0.05
BMI (kg/m^2^)	23.98±4.28	24.00±5.19	0.021	>0.05

### Treatment methods

The laparoscopic system was purchased from SONY. Automatic CO_2_ pneumoperitoneum machine was bought from Stryker Corporation (USA). High-frequency electrotome was used for incision and hemostasis. Surgeries for the two groups were performed by the same surgeon or two comparably competent surgeons, after which routine analgesia was carried out.

### Treatment group

This group received endoscopic thyroidectomy through the anterior chest wall via the areolar approach. In the supine position, one assistant stood their holding the laparoscope, and other assistants and nurses stood on the right. Three puncture trocars were placed at bilateral upper edges of the areola and beside the right sternum in the nipple-nipple line. Operating space was routinely established under the skin of the anterior chest wall and the platysma muscle, into which the trocar was inserted and CO_2_ was infused. lacunar cord-like connective tissues were sharply dissected until the sternal upper concave with an ultrasonic harmonic scalpel. Fascial surface of the sternocleidomastoid muscle was reached through separation along the smaller clavicular head, from which that of deep neck flexors and related loose tissues were sharply dissected.

Then thyroid cysts were exposed and resected. The thyroid pseudo-membrane was cut open and bluntly dissected to expose thyroid lobes on the cyst side. Afterwards, anterior thyroid muscle groups were pulled outward by using a self-made looped stitch to expose veins that were transected, during which the recurrent laryngeal nerve was protected from accidental injury. The surgical field was then hemostasized and rinsed, and the resected samples were sent for pathological examination. Finally, the surgical site was interruptedly sutured with 3-0 absorbable sutures, and drainage was routinely performed.

### Control group

Conventional open thyroidectomy was conducted. In the supine position, patient lied down with the head completely extended backwards to expose the neck and thyroid glands. An incision lengthed about 5-8 cm was made two fingerbreadths from the suprasternal notch to cut open the skin and subcutaneous tissues, and to separate loose connective tissues of bilateral sternocleidomastoid muscles and infrahyoid muscles until the upper and lower planes of side thyroid lobes. The linea alba cervicalis was then cut open to expose thyroid glands with lesions. Main thyroid blood vessels were ligated, and thyroid masses were separated and resected. Subsequently, the patient was treated with the same method as that for the treatment group.

### Observation indices

Measurement of thyroid hormone levels: Fasting venous blood (2 ml each tube) was drawn in the early morning before as well as one day and five days after surgery, and subjected to hematological examination within 24 h. Thyroid hormone indices, including FT3, FT4 and TSH, were detected by Roche E170 automatic electrochemiluminescence immunoassay analyzer.

### Determination of immune indices

The same venous blood samples were anticoagulated with heparin, treated by direct fluorescent labeling and detected by flow cytometry after dissolution of red blood cells, dilution and fixing. Percentages of NK, CD3+ T, CD4+ T and CD8+ T cells were detected by EPICS-XL flow cytometer (Coulter, USA).

### Measurement of serum calcium and phosphorous levels

The same venous blood samples were anti-coagulated with heparin, from which serum was separated to detect calcium and phosphorous levels by using Hitachi automatic biochemical analyzer (Japan). All patients were followed up for three months to observe adverse reactions, including surgical site infection, recurrent laryngeal nerve injury, parathyroid crisis, hematomas and hoarseness.

### Statistical analysis

All data were analyzed by SPSS17.0. The categorical data were expressed as mean ± standard deviation (x±s) and compared by repeated measures analysis of variance data, LSD method, non-parametric test and t test. The numerical data were compared by uncorrected and corrected χ^2^ tests. P<0.05 was considered statistically significant.

## RESULTS

### Changes of thyroid hormone levels

The two groups had similar thyroid hormone levels before surgery (P>0.05). After surgery, FT3 and FT4 levels significantly increased in both groups, whereas that of TSH significantly decreased (P<0.05). Meanwhile, the levels of the two groups differed significantly on the postoperative 5th day (P<0.05) (Table-II).

**Table-II:** Changes of thyroid hormone levels (x±s).

Group	Case number (n)	Before surgery	Postoperative 1st day	Postoperative 5th day
FT3-Treatment group	46	2.86±0.45	2.94±0.53*	3.17±0.45*
Control group	46	2.87±0.38	2.97±0.48*	4.09±0.56*#
FT4-Treatment group	46	12.47±1.76	18.09±2.98*	15.38±2.00*
Control group	46	12.51±2.87	18.16±4.22*	18.67±3.22*#
TSH-Treatment group	46	1.98±0.74	0.92±0.64*	0.84±0.55*
Control group	46	1.99±0.67	0.91±0.38*	0.93±1.23*#

* Compared with the levels before surgery, P<0.05;

# inter-group comparison, P<0.05.

### Changes of immunological indices

NK%, CD3+%, CD4+% and CD8+%, which were similar in the two groups before surgery, significantly fluctuated on the postoperative 1st day in both groups (P<0.05). Such indices were basically recovered on the postoperative 5th day in the treatment group that had significantly different outcomes from those of the control group (P<0.05) (Table-III).

**Table-III:** Changes of immunological indices (x±s).

Group	Case number (n)	Before surgery	Postoperative 1st day	Postoperative 5th day
NK%-Treatment group	46	10.31±2.78	13.45±3.09*	8.09±3.11*
Control group	46	10.37±3.31	12.36±5.33*	9.45±4.23*#
CD3+%-Treatment group	46	76.33±5.98	68.21±5.09*	75.23±7.38*
Control group	46	76.39±6.12	69.37±6.32*	70.78±12.11*#
CD4+%-Treatment group	46	44.61±5.09	38.78±5.88*	46.33±5.09*
Control group	46	44.76±5.67	37.89±6.13*	41.66±6.33*#
CD8+%-Treatment group	46	33.28±6.49	37.68±13.22*	31.78±5.67*
Control group	46	33.78±6.04	36.88±8.20*	28.99±6.33*#

* Compared with the levels before surgery, P<0.05;

# inter-group comparison, P<0.05.

### Changes of serum calcium and phosphorus levels

The serum calcium and phosphorous levels of the two groups were similar before surgery. On the postoperative 1st and 5th days, however, the treatment group had significantly lower serum calcium level and significantly higher serum phosphorus level than those of the control group (P<0.05) (Table-IV).

**Table-IV:** Changes of serum calcium and phosphorus levels (mmol/L, x±s).

Group	Case number (n)	Before surgery	Postoperative 1st day	Postoperative 5th day
Serum calcium-Treatment group	46	2.81±0.83	1.91±0.56*#	1.84±0.67*#
Control group	46	2.80±0.78	2.44±0.67*	2.11±0.44*
Serum phosphorous-Treatment group	46	0.72±0.33	1.00±0.38*#	1.08±0.11*#
Control group	46	0.73±0.41	0.88±0.42*	0.92±0.34*

* Compared with the levels before surgery, P<0.05;

# inter-group comparison, P<0.05.

### Adverse reactions

The surgeries were successfully performed for all patients, without unexpected changes to open thyroidectomy. During three months of follow-up, the treatment group was significantly less prone to complications such as surgical site infection, recurrent laryngeal nerve injury, parathyroid crisis and hoarseness than the control group (P<0.05). In the meantime, all complications were mitigated after symptomatic treatment, without death cases (Table-V).

**Table-V:** Postoperative adverse reactions (n).

Group	Case No.	Surgical site infection	Recurrent laryngeal nerve injury	Parathyroid crisis	Hematoma	Hoarseness
Treatment group	46	1 (2.2%)	0 (0.0%)	0 (0.0%)	1 (2.2%)	1 (2.2%)
Control group	46	5 (10.9%)	3 (6.5%)	4 (8.7%)	1 (2.2%)	4 (8.7%)
χ^2^		6.398	7.399	12.874	0.000	4.852
P		<0.05	<0.05	<0.05	>0.05	<0.05

## DISCUSSION

Parathyroid cysts are common in clinical practice, the incidence rates of which have obvious regional differences. Threatening people of all ages, this disease is mostly found in females aged 20-50 years old.^12^ Given the lack of early typical clinical symptoms, it is often misdiagnosed. Parathyroid cysts are commonly manifested as hyperparathyroidism,^13^ and then changes in serum calcium and parathyroid hormone levels with disease progression. Elevated serum calcium level can lead to a series of pathological changes in the skeletal, urinary and digestive systems, occasionally mass-induced dysphagia, dyspnea and hoarseness. This disease, if not treated timely, may be developed into parathyroid cancer that invades surrounding organs and leads to distal metastasis and high mortality rate.^14^

At present, parathyroid cysts can be treated by surgery and drugs such as radioiodine and levothyroxine. The surgery can be easily conducted by separating and resecting the cysts, without requiring exploration of the parathyroid glands. Since most cysts are located at the posterior, inferior position of thyroid glands near the intersection between inferior thyroid artery and recurrent laryngeal nerve, the nerve should be well protected.^15^ Although laparoscopic thyroid cystectomy has been widely applied, it is assigned to a traumatic surgery rather than a minimally invasive one owing to long surgical time, large surgical field and intense postoperative pain.^16^

In contrast, endoscopic thyroidectomy through the anterior chest wall via the areolar approach, which benefits cyst exploration as well as decreases the possibility of recurrent laryngeal nerve injury, surgical traumas and complications. Moreover, this strategy barely affects beauty by not separating large areas of subcutaneous tissues. As a result, complications such as respiratory acidosis and subcutaneous emphysema are prevented, and patients diagnosed early also enjoy short surgical time. In this study, the surgeries were successfully carried out for all patients, without unexpected changes to open thyroidectomy. During three months of follow-up, the treatment group was significantly less prone to complications such as surgical site infection, recurrent laryngeal nerve injury, parathyroid crisis and hoarseness than the control group (P<0.05). Meanwhile, all complications were mitigated after symptomatic treatment, without death cases.

Human body is bound to be injured after surgery as a special form of trauma that necessarily activates adaptive changes of homeostatic regulatory systems of which immune response is particularly sensitive. Within a certain range, immune response is enhanced with enlarging surgical trauma,^17^ which is closely associated with early postoperative wound healing and recovery. Immune cells and molecules have been prove to participate in surgical wound healing that requires sufficient immunity to resist the invasion of external pathogens and microorganisms.^18^ As an organ-specific autoimmune thyroid disease, primary hyperparathyroidism mainly results from immune system disorders based on the interaction between genetic and environmental factors.

NK cells are the first natural defenses against infections and tumors, and mature peripheral blood T cells are represented by CD3+ ones. CD3 molecule mainly assists T cell antigen receptor to recognize the major histocompatibility complex (MHC)-antigenic determinantcomplex on antigen presenting cells (APCs). Decrease in CD3+ T cells suggests that mature T cells reduce, thereby directly weakening the recognition of MHC-antigenic determinantcomplex. CD4+ T cells can promote the proliferation and differentiation of B, T and other immune cells, while CD8+ T cells can inhibit the activation stage of immune response.^19^ NK%, CD3+%, CD4+% and CD8+%, which were similar in the two groups before surgery, significantly fluctuated on the postoperative 1st day in both groups (P<0.05). Such indices were basically recovered on the postoperative 5th day in the treatment group that had significantly different outcomes from those of the control group (P<0.05). Therefore, the endoscopic thyroidectomy mildly affected immune function, so the patients recovered more rapidly.

Thyroid glands, as the largest endocrine glands in human body, are solely capable of extracellularly storing considerable generated hormones that meet metabolic requirements.^20^ The two groups had similar thyroid hormone levels before surgery (P>0.05). After surgery, FT3 and FT4 levels significantly rose in both groups, whereas that of TSH significantly dropped (P<0.05). Meanwhile, the levels of the two groups differed significantly on the postoperative 5th day (P<0.05). We attributed continuous high FT3 and FT4 levels to release of copious thyroid hormones existing as gelatinous substances due to surgical incision and extrusion of thyroid tissues. In the meantime, more thyroid hormones were required under surgical stress. On the other hand, decreased TSH level may result from negative feedback inhibition induced by rising levels of the above two hormones. In general, this method hardly affected the metabolism of thyroid hormones by minimizing the effects of surgical trauma on human body.

Serum calcium and phosphorous levels, which are important indices for primary hyperparathyroidism, should be timely monitored during the perioperative period. Continuous and severe hypocalcemia generally means successful resection of tumors, which should be corrected through calcium supplement, but persistent high calcium level after surgery indicates poor prognosis. The serum calcium and phosphorous levels of the two groups were similar before surgery. On the postoperative 1st and 5th days, however, the treatment group had significantly lower serum calcium level and significantly higher serum phosphorus level than those of the control group (P<0.05).

In summary, treating primary hyperparathyroidism with endoscopic thyroidectomy through the anterior chest wall via the areolar approach can decrease the risks of complications and facilitate recovery of serum calcium and phosphorous levels, probably by not evidently affecting immune function or thyroid hormones.

## References

[1] Traibi A, El-Hammoumi M, El-Oueriachi F, Arsalane A, Kabiri EH (2012). Benign cysts of the mediastinum: Series of 28 cases. Rev Mal Respir.

[2] Korpi-Hyövälti E, Cranston T, Ryhänen E, Arola J, Aittomäki K, Sane T (2014). CDC73 Intragenic Deletion in Familial Primary Hyperparathyroidism Associated With Parathyroid Carcinoma. J Clin Endocrinol Metab.

[3] Lam UD, Lerchbaum E, Schweighofer N, Trummer O, Eberhard K, Genser B (2014). Association of MEP1A gene variants with insulin metabolism in central European women with polycystic ovary syndrome. Gene.

[4] Hoi WH, Leow MK, Sule A, Lee HY, Mmed TA, Tay JC (2011). Hyperparathyroidism due to eutopic PTH secretion from an ectopic intrathymic parathyroid cyst. Ann Thorac Cardiovasc Surg.

[5] Ferrè S, Bongers EM, Sonneveld R, Cornelissen EA, van der Vlag J, van Boekel GA (2013). Early development of hyperparathyroidism due to loss of PTH transcriptional repression in patients with HNF1βmutations?. J Clin Endocrinol Metab.

[6] Dell'Amore A, Asadi N, Bartalena T, Bini A, Stella F (2014). Thoracoscopic resection of a giant mediastinal parathyroid cyst. Gen Thorac Cardiovasc Surg.

[7] Kim MY, Chung CY, Kim JS, Myung DS, Cho SB, Park CH (2013). Parathyroid cyst presenting as acute pancreatitis: report of a case. Chonnam Med J.

[8] Mincione G, Tarantelli C, Vianale G, Di Marcantonio MC, Cotellese R, Francomano F (2014). Mutual regulation of TGF-β1, TβRII and ErbB receptors expression in human thyroid carcinomas. Exp Cell Res.

[9] Karagkounis G, Chute DJ, Nasr CE, Berber E (2014). Tall-cell variant papillary thyroid carcinoma arising from struma ovarll. Endocr Pract.

[10] Kojima Y, Kamimura N, Yamamoto H, Murasawa H, Okamoto A, Imai A (2013). Successful control of hyper-cortisolemia due to ACTH-producing thyroid carcinoid by laparoscopic bilateral adrenalectomy: a case report. Hinyokika Kiyo.

[11] Vicente DA, Solomon NP, Avital I, Henry LR, Howard RS, Helou LB (2014). Voice outcomes after total thyroidectomy, partial thyroidectomy, or non-neck surgery using a prospective multifactorial assessment. J Am Coll Surg.

[12] Christensen MH, Pedersen EK, Nordbø Y, Varhaug JE, Midttun Ø, Ueland PM (2012). Vitamin B6 status & interferon-γ-mediated immune activation in primary hyperparathyroidism. J Intern Med.

[13] Kittaka A, Saito N, Takano M (2006). Recent progress of study on vitamin D analogs. Clin Calcium.

[14] Lee J, Chung WY (2013). Robotic thyroidectomy & neck dissection: past, present, & future. Cancer J.

[15] Sakoda M, Ueno S, Iino S, Minami K, Ando K, Kawasaki Y (2013). Pure laparoscopic subsegmentectomy of the liver using a puncture method for the target portal branch under percutaneous ultrasound with artificial ascites. Surg Laparosc Endosc Percutan Tech.

[16] Song CM, Cho YH, Ji YB, Jeong JH, Kim DS, Tae K (2013). Comparison of a gasless unilateral axillo-breast & axillary approach in robotic thyroidectomy. Surg Endosc.

[17] Liozon E, Loustaud-Ratti V, Soria P, Bezanahary H, Fauchais AL, Nadalon S (2004). Disease associations in 250 patients with temporal (giant cell) arteritis. Presse Med.

[18] Triggiani V, Resta F, Giagulli VA, Iovino M, Licchelli B, De Pergola G (2013). Parathyroid hormone determination in ultrasound-guided fine needle aspirates allows the differentiation between thyroid & parathyroid lesions: our experience & review of the literature. Endocr Metab Immune Disord Drug Targets.

[19] Lang CL, Wang MH, Hung KY, Chiang CK, Lu KC (2013). Altered molecular repertoire of immune system by renal dysfunction in the elderly: is prediction & targeted prevention in the horizon?EPMA J.

[20] Cañas CA, Bonilla-Abadía F, Anaya JM, Tobón GJ (2013). Is primary hyperparathyroidism a pathogenic factor in some conditions mediated by B lymphocytes hyperactivity?Med Hypotheses.

